# Programmable Organic A_2_S-TT NPs for NIR-II/Photoacoustic Imaging-Enabled Precision Therapy

**DOI:** 10.3390/pharmaceutics18091144

**Published:** 2026-09-10

**Authors:** Wei Sang, Yijun Huang, Jiahui Wen, Weiqing Yue, Ajing Wu, Jie Su, Ziliang Zheng, Jie Li, Ruiping Zhang

**Affiliations:** 1 Institute of Medical Technology, School of Basic Medical Sciences, Shanxi Medical University, Taiyuan 030001, China; 13313846608@163.com (J.W.); 17200588310@163.com (A.W.); 19834730881@163.com (J.S.); 2 The First Clinical Medical School, Shanxi Medical University, Taiyuan 030001, China; huangyijun2024@126.com; 3 State Key Laboratory of Flexible Electronics (LoFE), Institute of Advanced Materials (IAM), School of Flexible Electronics (Future Technologies), Nanjing Tech University (NanjingTech), Nanjing 211816, China; iamwqyue@njtech.edu.cn (W.Y.); iamjli@njtech.edu.cn (J.L.); 4 Laboratory of Molecular Imaging, Fifth Hospital of Shanxi Medical University (Shanxi Provincial People’s Hospital), Taiyuan 030012, China; zzlsxty@126.com

**Keywords:** triple-negative breast cancer, near-infrared II, photoacoustic imaging, photodynamic therapy

## Abstract

**Background**: Triple-negative breast cancer (TNBC) is a highly aggressive subtype of breast cancer characterized by significant treatment challenges and poor prognosis. Traditional therapies such as surgical resection, radiotherapy, and chemotherapy often suffer from limitations including insufficient therapeutic specificity, potential damage to normal tissues, and limited imaging capability. The integrated diag{Pareja, 2018 #13}nosis-treatment platform combines diagnostic and therapeutic functions, offering a novel approach for real-time tumor monitoring and precision therapy. **Methods**: Accordingly, this study designed and developed an integrated diagnostic and therapeutic platform called A_2_S-TT NPs, utilizing self-assembled nanoparticles incorporating organic conjugated molecules for precise photodynamic therapy (PDT) guided by dual modalities of Near-infrared II (NIR-II) fluorescence imaging and photoacoustic imaging (PA). **Results**: Under 808 nm laser excitation, A_2_S-TT NPs efficiently generated reactive oxygen species (ROS) at the tumor site, inducing mitochondrial membrane potential disruption and DNA damage to activate apoptosis. In vitro experiments demonstrated that A_2_S-TT NPs exhibited intense NIR-II fluorescence and photoacoustic signals, showcasing excellent dual-modal imaging potential. Moreover, it demonstrated minimal cytotoxicity in the absence of light exposure but significantly enhanced tumor cell killing under laser irradiation, demonstrating superior biosafety and photodynamic efficacy. In vivo studies further confirmed that A_2_S-TT NPs effectively accumulated at tumor sites, enabling visualization-based monitoring through its mediated NIR-II and PA dual-modal imaging. Additionally, A_2_S-TT NPs-mediated PDT exhibited remarkable tumor-suppressive effects while avoiding significant damage to normal tissues. **Discussion**: This study constructs a novel PA/NIR-II dual-modal imaging-guided PDT platform based on donor–acceptor conjugated polymer A_2_S-TT NPs for integrated TNBC theranostics. PA provides high-resolution deep-tissue imaging, while NIR-II fluorescence offers high sensitivity and deep penetration; their complementary strengths support precise tumor localization and real-time therapeutic monitoring. Under dual-modal guidance, A_2_S-TT NPs enrich in tumors and generate abundant ROS upon 808 nm laser irradiation to boost PDT efficacy. In vitro and in vivo biosafety tests confirm its favorable biocompatibility with minimal systemic toxicity at therapeutic doses. **Conclusions**: In conclusion, A_2_S-TT NPs achieved precise, efficient, safe, and controllable PDT through dual-mode imaging using NIR-II and PA, providing a novel strategy with clinical translation potential for the integrated diagnosis and treatment of TNBC.

## 1. Introduction

TNBC is one of the most aggressive and most poorly prognosed molecular subtypes of breast cancer, accounting for approximately 15–20% of all breast cancer cases [1,2]. Due to the absence of expression of estrogen receptors, progesterone receptors, and human epidermal growth factor receptor 2 (HER2), TNBC patients cannot benefit from endocrine therapy or HER2-targeted therapy and are prone to distant metastasis and shortened overall survival, despite continuous advancements in comprehensive treatment strategies—including surgery, radiotherapy, chemotherapy, and immunotherapy [3]. In recent years, the overall prognosis of TNBC patients has not fundamentally improved [4]. The primary reasons include high tumor heterogeneity, lack of effective therapeutic targets, and insufficient precision of conventional treatments, which often cause damage to normal tissues and significant toxic side effects. Therefore, developing novel diagnostic and therapeutic approaches that combine precise diagnosis with efficient treatment is crucial for improving clinical outcomes in TNBC patients.

Currently, imaging remains a crucial tool for early breast cancer screening, lesion localization, and therapeutic efficacy evaluation [5,6]. However, existing imaging techniques all exhibit certain limitations. Ultrasonography offers advantages such as ease of operation and low cost, but its ability to detect minute lesions and deep tissues is limited. Mammography is an essential method for breast cancer screening; however, it relies on ionizing radiation and demonstrates relatively low sensitivity for dense-type breast cancers. Although magnetic resonance imaging (MRI) exhibits excellent soft tissue differentiation capability, it requires prolonged examination time, incurs high costs, and is constrained by certain clinical conditions [7,8]. More importantly, traditional imaging techniques primarily serve diagnostic purposes and are inadequate for real-time monitoring or dynamic feedback during treatment. Therefore, developing novel imaging technologies that combine high-sensitivity, deep tissue visualization, and treatment navigation capabilities represents a key direction for achieving precision diagnosis and treatment of breast cancer.

In recent years, the integration of diagnosis and therapy has provided novel approaches for the advancement of precision oncology. By combining diagnostic and therapeutic functions on a single nanoscale platform, this integrated approach enables precise lesion localization prior to treatment, real-time monitoring during therapy, and post-treatment efficacy evaluation [9,10]. With the rapid development of nanomedicine, an increasing number of multifunctional nanosystems have been applied in areas such as tissue repair, inflammation regulation, and cancer treatment, demonstrating promising clinical applications [11,12]. However, for TNBC, there remains a lack of multifunctional diagnostic and therapeutic platforms that simultaneously achieve deep tissue imaging, high-sensitivity localization, and localized precision therapy. Therefore, developing novel nanodiagnostic and nanotherapeutic systems that integrate high-performance imaging with targeted therapy remains a critical focus in current nanomedicine research [13].

NIR-II fluorescence imaging and PA imaging have garnered significant attention due to their complementary advantages [14,15]. NIR-II fluorescence imaging features low tissue scattering, weak autofluorescence, deep tissue penetration, and high signal-to-noise ratios, enabling highly sensitive imaging of deep tissues [16,17]. PA imaging combines optical excitation with ultrasound detection, offering both high tissue penetration depth and spatial resolution, while providing functional information such as tumor vasculature, blood oxygen levels, and nanomaterial distribution [18,19]. The combination of these two techniques leverages the high-sensitivity detection capabilities of NIR-II and the high-resolution imaging properties of PA, delivering comprehensive informational support for precise tumor localization, treatment navigation, and therapeutic efficacy evaluation [7,15,20].

PDT is recognized as a highly promising precision oncology strategy due to its advantages of being minimally invasive, spatiotemporally controllable, and repeatable [21,22]. It achieves localized tumor ablation by inducing ROS through photosensitizers excited at specific wavelengths, thereby triggering oxidative stress, mitochondrial dysfunction, and apoptosis. However, conventional PDT is limited by inadequate tumor localization and the lack of real-time imaging feedback during treatment. Therefore, integrating PDT with high-performance imaging technologies to establish an imaging-guided integrated diagnosis-treatment platform represents a critical direction for enhancing therapeutic precision [23,24].

Building upon this concept, we designed and developed an integrated PDT and diagnosis platform (A_2_S-TT NPs) utilizing NIR-II/PA dual-modal imaging for precise diagnosis and treatment of TNBC. This platform organically integrates NIR-II fluorescence imaging, PA, and PDT within a single nanosystem. By leveraging the complementary advantages of dual-modal imaging in tumor localization, deep tissue visualization, and dynamic monitoring during treatment, it enables imaging-guided precision PDT. The A_2_S-TT NPs nanostructure serves as the core functional scaffold, efficiently generating ROS under 808 nm laser irradiation to induce tumor cell apoptosis, thereby achieving efficient and localized ablation of tumor tissues. In this study, the 4T1 cell line, a well-established TNBC cell line whose tumor growth, invasion, and metastasis in BALB/C mice closely mimic human breast cancer, was employed as the cellular model for both in vitro and in vivo evaluations. We systematically evaluated the physicochemical properties, optical performance, ROS generation capacity, NIR-II/PA dual-modal imaging performance, and photodynamic efficacy of the platform both in vitro and in vivo, further validating its biosafety, aiming to provide novel insights for the design and clinical translation of integrated nanoplatforms for TNBC diagnosis and treatment.

## 2. Materials and Methods

### 2.1. Materials

Methanol (A030092) was obtained from Energy Chemical (Shanghai, China). Dichloromethane (D116144) and dimethyl sulfoxide (DMSO, D103281) were purchased from Aladdin Biochemical Technology Co., Ltd. (Shanghai, China). Toluene (503021204-500mL) was supplied by Yonghua Chemical Co., Ltd. (Suzhou, China). THF (T818767-500mL) was obtained from Macklin Biochemical Co., Ltd. (Shanghai, China). TT (IN1172-5g) was purchased from Suzhou Nakai Chemical Co., Ltd. (Suzhou, China), and AS_2_ (ZY241853) from Nanjing Zhiyan Biotechnology Co., Ltd. (Nanjing, China). Pd(PPh_3_)_2_Cl_2_ (242195) was acquired from J&K Scientific (Beijing, China). Pluronic F-127 (768502) and 1,3-diphenylisobenzofuran (D808285) were acquired from Macklin Biochemical Co., Ltd. (Shanghai, China). Absolute ethanol (E99945) was obtained from Acme (Beijing, China). 4% paraformaldehyde solution, DMEM high-glucose medium, RPMI-1640 medium, penicillin-streptomycin solution (PS), PBS, trypsin-EDTA solution, CCK-8 assay kit, JC-1 mitochondrial membrane potential detection kit, 2′,7′-Dichlorodihydrofluorescein diacetate (DCFH-DA) reactive oxygen species fluorescent probe, Calcein-AM/PI live/dead cell double staining kit, TUNEL apoptosis detection kit, and H&E staining kit were purchased from Jiangsu KeyGen Biotech Co., Ltd. (Nanjing, China). Anti-γ-H2AX antibody (AF5836), anti-Ki-67 antibody, anti-CRT antibody, anti-HMGB1 antibody, Triton X-100 (P0096-100), anti-fade fluorescence mounting medium, and 594-conjugated secondary antibody (A1200-100) were obtained from Beyotime Biotechnology (Shanghai, China). Chloroform, 5-amino-1-pentanol, 1,4-butanediol diacrylate, 4′,6-diamidino-2-phenylindole, polyethylenimine, 1,1′-dioctadecyl-3,3,3′,3′-tetramethylindotricarbocyanine iodide, phosphotungstic acid staining solution, xylene, neutral balsam, EDTA antigen retrieval solution, and DAB chromogenic kit were used in this work. All other reagents and solvents were of analytical grade and used without further purification.

### 2.2. Synthesis and Characterization of A_2_S-TT NPs Conjugated Polymer by Gel Permeation Chromatography (GPC) and Nuclear Magnetic Resonance (NMR)

The target conjugated polymer AT was synthesized by a Stille polycondensation reaction, using the distannylated monomer TT and the dibrominated acceptor monomer AS_2_ as building blocks. The reaction was carried out in anhydrous toluene at 85 °C with Pd(PPh_3_)_2_Cl_2_ as the catalyst.

Specifically, the synthesis was conducted as follows. A two-neck round-bottom flask was charged with the distannylated monomer, (4,8-bis(5-(2-ethylhexyl)-4-chlorothiophen-2-yl)benzodithiophene-2,6-diyl)-bis-(trimethylstannane) (TT) (180.9 mg, 0.2 mmol), and the dibrominated monomer, 2,2′-[[12,13-Dihydro-12,13-bis(2-octyldodecyl)-3,9-diundecylbisthieno [2″,3″:4′,5′]thieno [2′,3′:4,5]pyrrolo [3,2-e:2′,3′-g][2,1,3]benzothiadiazole-2,10-diyl]bis[methylidyne(bromo-3-oxo-1H-indene-2,1(3H)-diylidene)]]bis[propanedinitrile] (AS_2_) (187.4 mg, 0.1 mmol). Subsequently, anhydrous toluene (1 mL) was added to dissolve the monomers. The solution was purged with nitrogen to remove oxygen. Then, the catalyst, bis(triphenylphosphine)dichloropalladium(II) (Pd(PPh_3_)_2_Cl_2_), was added under a nitrogen atmosphere.

The reaction mixture was heated to 85 °C and stirred for 3.5 h under a nitrogen atmosphere. After the reaction was completed, the mixture was allowed to cool to room temperature. The crude product was filtered through an organic filter membrane to remove the solid catalyst residues. The filtrate was then concentrated and purified by precipitation into methanol. The resulting precipitate was collected by filtration and dried under vacuum, yielding the target polymer AT as a dark solid (225 mg, 60% yield).

The molecular weight of AT was determined by GPC using tetrahydrofuran as the mobile phase and polystyrene standards for calibration. The polymer exhibited an Mr of 7341 g/mol, a weight-average molecular weight Mw of 14,267 g/mol, with a PDI of 1.94. The chemical structure was further confirmed by ^1^H NMR spectroscopy.

### 2.3. FTIR Spectroscopy Characterization of A_2_S-TT NPs

A small amount of the sample AS_2_, TT, and AT was mixed with dry spectroscopic-grade KBr in a 1:100 ratio. The mixture was thoroughly ground in an agate mortar and then dried under an infrared lamp. The resulting powder was placed into a pellet die and pressed into a transparent thin film under vacuum pressure. The Fourier transform infrared spectrometer (INVENIO-S, Bruker, Ettlingen, Germany) was preheated for 30 min, with the scanning range set from 4000 to 400 cm^−1^. After collecting the KBr blank background spectrum, the sample pellet was scanned to obtain the infrared absorption spectrum, followed by baseline correction and peak position labeling.

### 2.4. TEM and Elemental Mapping Characterization of A_2_S-TT NPs

The A_2_S-TT NPs dispersion was diluted with double-distilled water and briefly sonicated to achieve a homogeneous dispersion. A carbon-coated copper TEM grid was placed on filter paper, with its carbon side facing upward. Approximately 20 μL of the suspension was deposited onto the grid surface. After air-drying under ambient conditions, TEM was used to analyze the morphological features and elemental mapping of the as-prepared A_2_S-TT NPs. The particle size was then statistically analyzed based on the TEM micrographs.

### 2.5. Hydrodynamic Diameter Measurement of A_2_S-TT NPs

The A_2_S-TT NPs solution was diluted with double-distilled water and sonicated in an ultrasonic water bath for 5 min to achieve uniform dispersion. Once fully dispersed, 1 mL of the A_2_S-TT NPs solution was transferred to a particle size cuvette. The hydrodynamic diameter of the A_2_S-TT NPs was determined using dynamic light scattering (DLS) with a Zetasizer Advance system (Malvern Panalytical Ltd., Malvern, UK). Each measurement was independently repeated three times, and the average hydrodynamic diameter was reported.

### 2.6. Zeta Potential Measurement of A_2_S-TT NPs

The A_2_S-TT NPs solution was diluted with double-distilled water and sonicated in an ultrasonic water bath for 5 min to achieve uniform dispersion. Once fully dispersed, 1 mL of the A_2_S-TT NPs solution was transferred to the particle size measurement cuvette. The Zeta potential of A_2_S-TT NPs was measured using the Zetasizer Advance system (Malvern Panalytical Ltd., Malvern, UK) by the capillary method. Each measurement was independently repeated three times, and the final report presented the average zeta potential test results.

### 2.7. Colloidal Stability Assessment of A_2_S-TT NPs

A_2_S-TT NPs were dispersed uniformly in PBS via ultrasonication and stirred to ensure a homogeneous suspension. The particle size and potential changes were measured using a Zetasizer Advance system (Malvern Panalytical Ltd., Malvern, UK) at 0, 2, 4, 8, 12, and 24 h. The suspension was monitored for signs of aggregation, precipitation, or visible changes such as color or turbidity.

A_2_S-TT NPs were uniformly dispersed in four media (water, PBS, DMEM, and DMEM + PBS) using ultrasonication, followed by stirring to ensure a homogeneous suspension. The particle size and potential changes were measured using a Zetasizer Advance system (Malvern Panalytical Ltd., Malvern, UK). The suspension was monitored for signs of aggregation, precipitation, or visible changes such as color or turbidity.

### 2.8. Optical Properties of A_2_S-TT NPs Evaluated by UV-Vis Spectroscopy

The UV-vis absorption spectrum of A_2_S-TT NPs was obtained through UV-vis spectroscopy. First, A_2_S-TT NPs were dispersed in Pluronic F127 and dissolved in double-distilled water to prepare a stable suspension. A fixed volume of the solution was then pipetted into a cuvette. The suspension was then analyzed using a UV-vis spectrophotometer across the wavelength range of 400–1000 nm, with absorbance values recorded at each wavelength to generate the UV-vis absorption spectrum.

### 2.9. In Vitro ROS Generation Capability of A_2_S-TT NPs Using DPBF Decomposition

The photodynamic activity of A_2_S-TT NPs was evaluated by monitoring the decomposition of 1,3-Diphenylisobenzofuran (DPBF) under 808 nm laser irradiation. A_2_S-TT NPs were first dispersed in Pluronic F127 and dissolved in double-distilled water to prepare a stable suspension, while DPBF was dissolved in Dimethylformamidex (DMF) to obtain a working solution. An aliquot of the A_2_S-TT NPs solution was mixed with the DPBF working solution and transferred to a quartz cuvette. The mixture was then exposed to an 808 nm laser at a power density of 1.5 W/cm^2^ for incremental time intervals of 0, 6, 12, 30, 60, and 120 s. UV-vis absorption spectra of the mixture were recorded using a UV-Vis spectrophotometer over the wavelength range of 400–1100 nm. The relative photodynamic efficacy and ROS generation capacity were evaluated using DPBF as an indicator.

### 2.10. NIR-II Fluorescence Imaging and Concentration-Intensity Linearity of A_2_S-TT NPs

A stock solution of A_2_S-TT NPs was first prepared in double-distilled water at a high concentration. Subsequent working solutions were then obtained by serial dilution to achieve final concentrations of 7.8125 μg/mL, 15.625 μg/mL, 31.25 μg/mL, 62.5 μg/mL, 125 μg/mL, and 250 μg/mL. For each concentration, 500 μL of solution was transferred into a 1.5 mL microcentrifuge tube, and fluorescence signals were acquired using a small animal NIR-II fluorescence in vivo imaging system. Imaging was performed in triplicate for each concentration, with fluorescence intensities recorded and corresponding images stored.

### 2.11. PA Signal Intensity of A_2_S-TT NPs

A stock solution of A_2_S-TT NPs was prepared in double-distilled water at a high concentration and was then serially diluted to obtain working solutions with final concentrations of 125 μg/mL, 250 μg/mL, 500 μg/mL, 750 μg/mL, and 1000 μg/mL. Double-distilled water was used as the control sample and loaded into the LOIS-3D optoacoustic imaging system (TomoWave Laboratories, Inc., Houston, TX, USA). The photoacoustic signal intensity was measured across the wavelength range of 700–850 nm. PA images corresponding to each concentration were generated using imaging software, and the correlation between signal intensity and A_2_S-TT NPs concentration was analyzed using linear regression.

### 2.12. 4T1 Murine Breast Cancer Cell Culture Procedures

The complete medium was prepared by combining high-glucose DMEM, 10% FBS, and 1% PS. 4T1 cells were uniformly seeded into culture flasks containing the complete medium and incubated at 37 °C with 5% CO_2_. When the cells reached 80–90% confluence, the medium was discarded, and the cells were washed with sterile PBS to remove residual medium. After washing, 0.25% trypsin-EDTA solution was added to detach the cells, which were incubated at 37 °C with 5% CO_2_. for 1–2 min until complete detachment was observed. Complete medium was added to neutralize the trypsin, followed by gentle pipetting to form a single-cell suspension. The cell suspension was then split at a 1:4 ratio into new flasks and incubated for further subculture.

### 2.13. Cellular Uptake Behavior of A_2_S-TT NPs in 4T1 Cells

The 4T1 cells were seeded at a density of 1 × 10^4^ cells per well into a 12-well plate and cultured in a 37 °C incubator. After 24 h, when the 4T1 cells had fully adhered, the original culture medium was removed and replaced with DMEM containing A_2_S-TT NPs; the cells were then incubated in the dark at 37 °C for 0, 2, 4, 8 and 12 h, respectively. Upon reaching the designated incubation time, the old culture medium was promptly removed, and each well was washed three times with PBS. After removing the PBS, 1 mL of 4% paraformaldehyde was added to each well and the samples were fixed in the dark at 4 °C for 30 min, followed by further fixation in the dark at 37 °C for an additional 4-8 h. Upon completion of fixation, the 4% paraformaldehyde was removed, and the wells were washed three times with PBS. Finally, the samples were imaged using a small animal NIR-II system (Suzhou Yingrui Optical Technology Co., Ltd., Suzhou, China).

### 2.14. CCK-8 Assay for Photodynamic Cytotoxicity and IC_50_ Measurement

4T1 cells were divided into three groups, with G1 as the control group, G2 as A_2_S-TT NPs alone, and G3 as A_2_S-TT NPs plus laser irradiation, and each group had six replicate wells. Cells were seeded into 96-well plates at a density of 1 × 10^3^ cells per well, with six replicates for each group, and incubated for 24 h to allow adhesion and stabilization. After confirming optimal cell viability, the medium was removed. G1 received 100 μL of DMEM medium without A_2_S-TT NPs. G2 and G3 received 100 μL of DMEM containing A_2_S-TT NPs at varying concentrations. After 24 h, the medium was replaced with fresh DMEM, and G3 was subjected to 808 nm laser irradiation, followed by an additional 12 h incubation. Subsequently, 10 μL of CCK-8 reagent was added to each well, including three blank wells containing only medium and CCK-8 without cells. After 30 min of incubation, the absorbance at 450 nm was measured using a microplate reader. Cell viability was calculated, and GraphPad Prism 9.0.0 was used to determine the IC_50_ values.

(1) $$\mathrm{Cell}\;\mathrm{viability}\;(\%)=\left[\frac{{\mathrm{OD}}_{\mathrm{treat}}-{\mathrm{OD}}_{\mathrm{blank}}}{{\mathrm{OD}}_{\mathrm{negative}\;\mathrm{control}}-{\mathrm{OD}}_{\mathrm{blank}}}\right]\times 100\%$$

### 2.15. Immunofluorescence Detection of DNA Double-Strand Breaks Induced by A_2_S-TT NPs PDT

4T1 cells were divided into three groups: the control group without treatment, the group treated with A_2_S-TT NPs, and the group treated with A_2_S-TT NPs followed by laser irradiation. Each group included three replicate confocal culture dishes. Cells were seeded at 1 × 10^6^ cells per dish and incubated for 12 h to allow adhesion. For groups G2 and G3, complete medium containing 20 μg/mL of A_2_S-TT NPs was added, and the cells were incubated for another 12 h. Afterward, the medium was replaced with fresh medium, and group G3 was irradiated with an 808 nm laser at 200 mW/cm^2^ for 5 min. All cells were then fixed with 4% paraformaldehyde for 10 min and washed with PBS. Anti-γ-H2AX antibody (1:500) was added, and cells were incubated at 37 °C in the dark for 1 h. After washing with PBS, DAPI staining was performed for 5 min. The fluorescence intensity of γ-H2AX foci in each group was observed and recorded using a confocal microscope.

### 2.16. JC-1 Staining for Evaluating Mitochondrial Membrane Potential Depolarization After A_2_S-TT NPs PDT

4T1 cells were seeded in 6-well plates at a density of 1 × 10^6^ cells per well and divided into three groups: the control group without treatment (G1), the group treated with A_2_S-TT NPs (G2), and the group treated with both A_2_S-TT NPs and laser irradiation (G3). After 24 h, when the cells had attached completely, the plates were removed, and the culture medium was aspirated. Fresh DMEM was added to G1, while DMEM containing 20 μg/mL A_2_S-TT NPs was added to both G2 and G3. The plates were then incubated for another 24 h. After incubation, the medium was aspirated, and the cells were washed once with PBS. Then, 1 mL of fresh complete DMEM was added to each well. G3 was irradiated with an 808 nm laser at a power density of 200 mW/cm^2^ for 5 min. Following irradiation, JC-1 staining solution was prepared according to the manufacturer’s protocol. For all groups, 1 mL of JC-1 working solution was added to each well, mixed gently, and the plates were incubated for 20 min in the incubator. Pre-chilled Assay Buffer was used after the incubation period to wash the cells. After washing twice to remove excess staining, 2–3 mL of fresh complete DMEM was added to each well. Cells were imaged under a fluorescence microscope, and fluorescence intensity patterns (red/green ratios) were processed via image analysis. Statistical analysis was performed to evaluate mitochondrial membrane potential depolarization across the experimental groups.

### 2.17. Evaluation of Live/Dead Cell Viability After A_2_S-TT NPs PDT

4T1 cells were seeded into confocal culture dishes at a density of 1 × 10^6^ cells per well with 2 mL of complete medium. The experiment consisted of three groups: G1 (control), G2 (A_2_S-TT NPs), and G3 (A_2_S-TT NPs + laser irradiation). DMEM containing 20 μg/mL A_2_S-TT NPs was then prepared. After this incubation, the medium in group G1 was replaced with fresh DMEM, whereas groups G2 and G3 received the A_2_S-TT NPs-containing DMEM, and all groups were cultured for another 24 h. Subsequently, the medium was aspirated, the cells were washed once with PBS, and 1 mL of fresh medium was added to each group. Group G3 was exposed to 808 nm laser irradiation for 5 min at a power of 200 mW/cm^2^. Following irradiation, 5 μL of Calcein AM at 2 mM and 15 μL of PI solution at 1.5 mM were added to each group, and the cells were stained for 15 min in the dark. After three washes with PBS to remove excess dye, cellular images were observed and recorded using a confocal laser scanning microscope.

### 2.18. Intracellular ROS Detection After A_2_S-TT NPs PDT

DCFH-DA was diluted to a final concentration of 10 μM with serum-free DMEM and used to detect intracellular ROS generation after 808 nm laser irradiation for evaluating the in vitro photodynamic performance of A_2_S-TT NPs. DCFH-DA readily penetrates the cell membrane and is hydrolyzed by intracellular esterases to non-fluorescent DCFH, which is subsequently oxidized by intracellular ROS to generate the green fluorescent product DCF. Therefore, the fluorescence intensity of 2,7-Dichlorofluorescein (DCF) reflects the intracellular ROS level.

4T1 cells were seeded in 12-well plates at a density of 2 × 10^5^ cells per well and allowed to attach for 24 h. The culture medium was then replaced with fresh complete medium containing A_2_S-TT NPs (20 μg/mL) for the G2 and G3 groups, while the G1 group received fresh complete medium only. After incubation for 12 h, the G3 was irradiated with an 808 nm laser at a power density of 200 mW/cm^2^ for 5 min. Immediately after irradiation, the cells were washed three times with PBS and incubated with serum-free DMEM containing 10 μM DCFH-DA in the dark at 37 °C for 20 min. The cells were then washed three times with PBS, and fluorescence images were acquired using a fluorescence microscope under identical imaging settings. Mean fluorescence intensity was quantified using ImageJ software 1.5.4..

### 2.19. Experimental Animals and Housing Conditions

Female BALB/c mice aged 6–8 weeks were purchased from Beijing Vital River Laboratory Animal Technology Co., Ltd. (Beijing, China) and used in all animal experiments. The animals were housed under SPF conditions with a 12 h light/dark cycle at controlled temperature and humidity. The production license number of the experimental animals was SCXK (Jin) 2024-0004, and the laboratory animal facility license number was SYXK (Jin) 2024-0007.

### 2.20. In Vivo NIR-II Fluorescence Imaging and Biodistribution

To evaluate the in vivo tumor accumulation and biodistribution of A_2_S-TT NPs, a subcutaneous 4T1 tumor-bearing BALB/c mouse model was established. Mice were anesthetized with isoflurane and intravenously injected with A_2_S-TT NPs at 1 mg/mL in aqueous solution. Whole-body NIR-II fluorescence images were acquired at 0.25, 1, 6, 12, 24, and 48 h post-injection using a small animal NIR-II system (Suzhou Yingrui Optical Technology Co., Ltd.) equipped with an 808 nm excitation laser and a 1000 nm long-pass emission filter. Images were acquired with an exposure time of 10 ms and a laser power of 1 W. Fluorescence intensities were quantified using ImageJ software to generate the time-dependent tumor accumulation profile. At each designated time point, mice were euthanized after in vivo imaging. The heart, liver, spleen, lungs, kidneys, and tumors were harvested, rinsed with saline, blotted dry, and subjected to ex vivo NIR-II fluorescence imaging under the same acquisition parameters. Fluorescence intensities were analyzed using ImageJ to evaluate the tissue biodistribution of A_2_S-TT NPs.

### 2.21. In Vivo PA Imaging

To evaluate the tumor accumulation of A_2_S-TT NPs, a subcutaneous 4T1 tumor-bearing BALB/c mouse model was established. Mice were intravenously injected with A_2_S-TT NPs at a concentration of 1 mg/mL. PA was performed using a TomoWave LOIS-3D PA system. Mice were anesthetized with isoflurane and maintained under inhalational anesthesia throughout image acquisition. Cross-sectional photoacoustic images of the tumor were acquired at 1, 3, 6, 12, 24, and 48 h post-injection using an excitation wavelength of 808 nm. Photoacoustic signal intensities were quantified using ImageJ to evaluate the time-dependent tumor accumulation of A_2_S-TT NPs.

### 2.22. NIR-II/PA-Guided PDT and In Vivo Safety Evaluation

Female BALB/c mice bearing 4T1 breast tumors were established by subcutaneous injection of 2 × 10^4^ cells into the left axillary region under isoflurane anesthesia. When tumors reached the desired size, mice were randomly divided into three groups: (G1) PBS, (G2) A_2_S-TT NPs, and (G3) A_2_S-TT NPs + laser irradiation. A_2_S-TT NPs were administered via tail-vein injection on days 1, 4, 7, 10, and 13, followed by 808 nm laser irradiation on days 2, 5, 8, 11, and 14. Body weight and tumor dimensions were recorded every two days throughout the treatment period. Tumor volume was calculated using the standard formula.

(2) $$V_{\mathrm{tumor}}\;\left(mm^{3}\right)=\frac{L\times W^{2}}{2}$$

On day 15, mice were euthanized under deep isoflurane anesthesia, and tumors were excised, photographed, weighed, and measured. The tumor inhibition rate (TIR) was calculated according to the following equation.

(3) $$\mathrm{TIR}\;\left(\%\right)=\left(\frac{m_{1}-m_{2}}{m_{1}}\right)\times 100\%$$

For biosafety evaluation, the heart, liver, spleen, lungs, and kidneys were harvested, fixed in 4% paraformaldehyde, processed using standard paraffin-embedding procedures, sectioned, and stained with H&E. Histopathological examination was performed under a light microscope to evaluate the potential systemic toxicity of A_2_S-TT NPs.

## 3. Results

### 3.1. Design Strategy, Structural and Morphological Characterization of A_2_S-TT NPs

**Figure 1:**
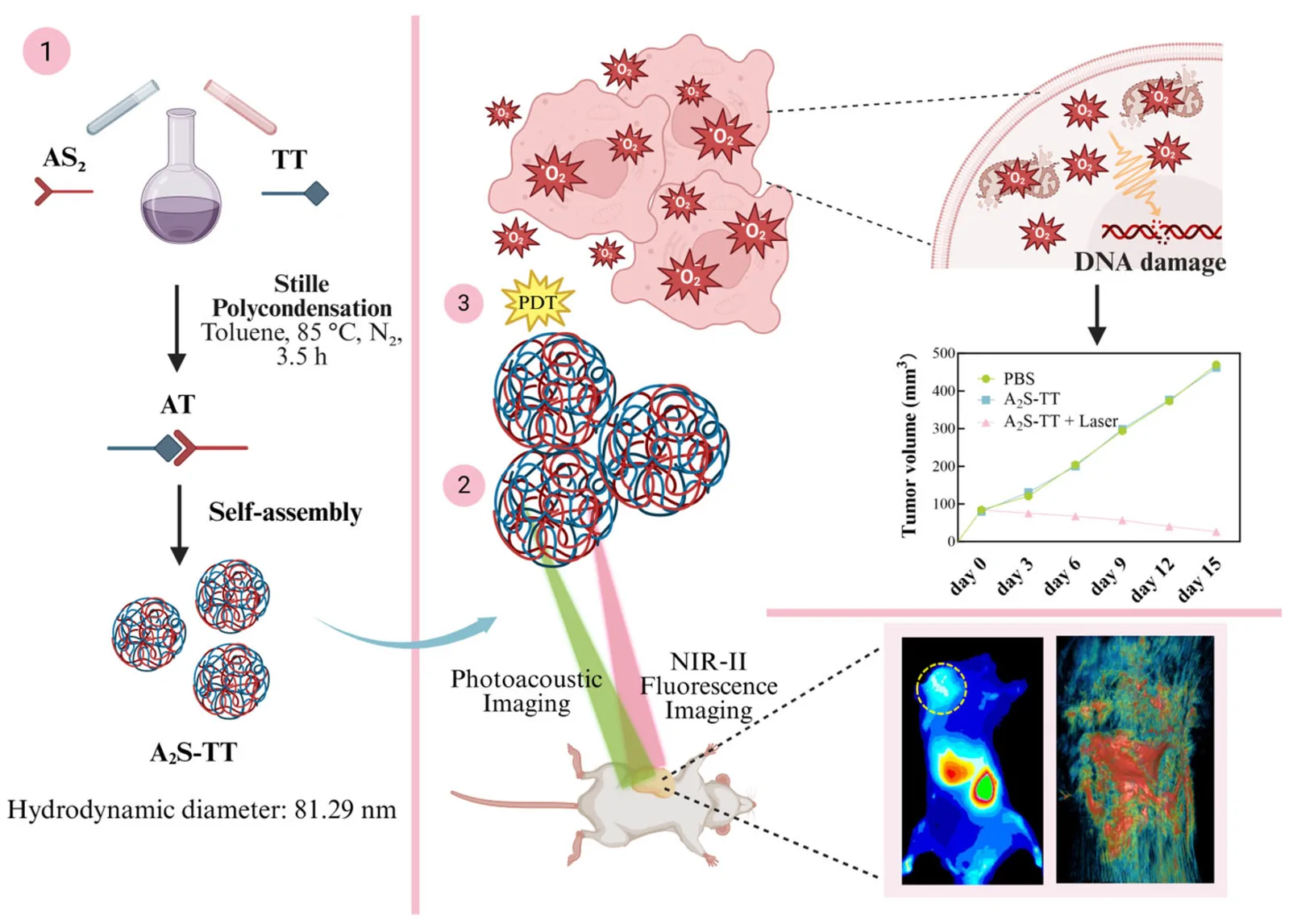
Schematic illustration of the synthesis, self-assembly, and multifunctional biomedical applications of A_2_S-TT NPs. The explanation for the numbers: 1: Preparation process of A_2_S-TT NPs; 2: Schematic diagram of NIR-II + PA-mediated multimodal imaging combined with A_2_S-TT NPs-based photodynamic therapy for breast cancer treatment in mice; 3: Photodynamic therapy efficacy. The explanation foe the colors: The yellow dashed circle indicates the tumor region; blue represents lower signal intensity, while red represents higher signal intensity.

The overall design strategy of the donor-acceptor conjugated polymer AT for multifunctional nanotheranostics construction is illustrated in Figure 1.

**Figure 2:**
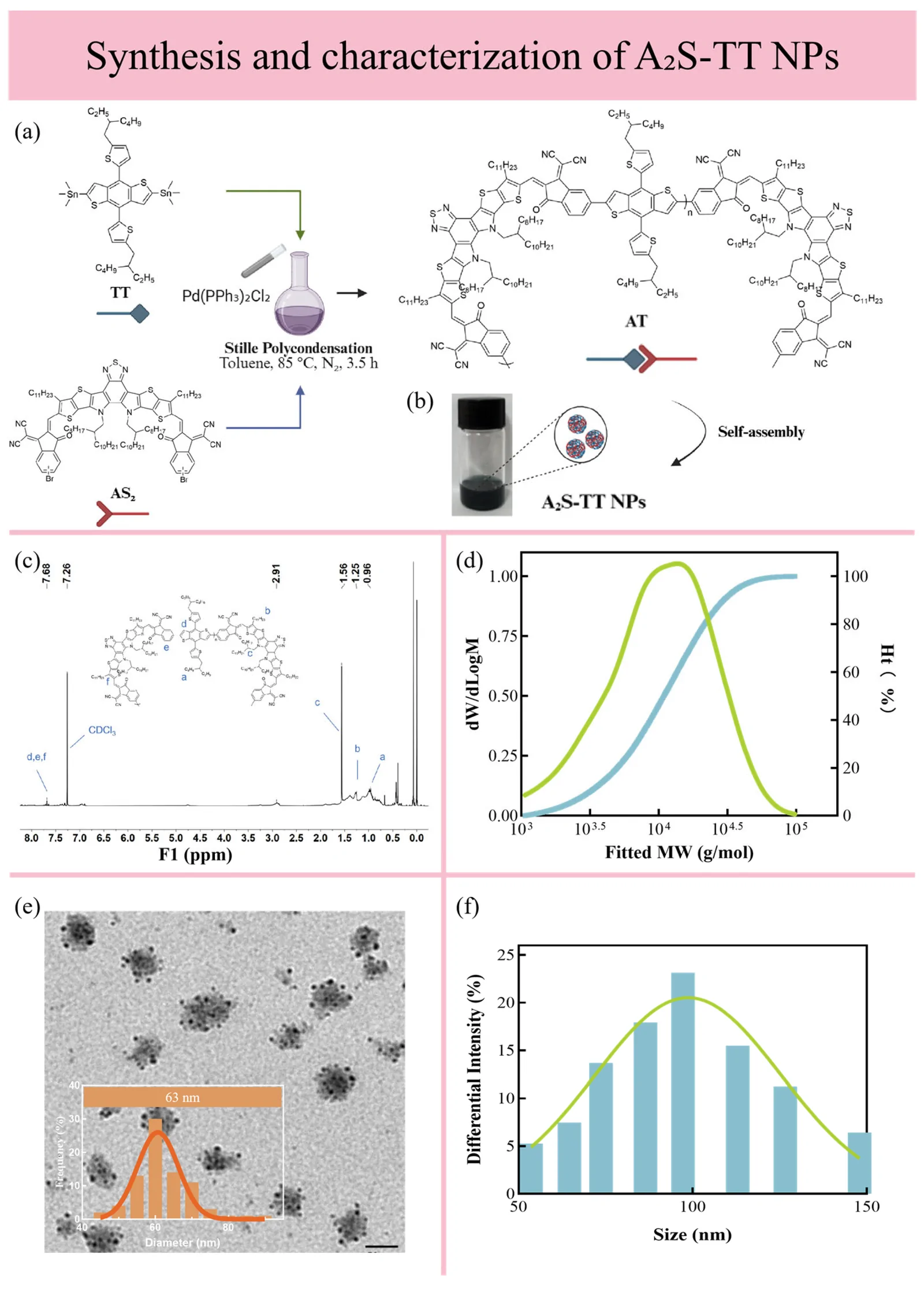
Fabrication and physicochemical characterization of A_2_S-TT NPs nanotheranostics. (**a**) Synthetic route schematic; (**b**) Picture of the real product (ink-black); (**c**) ^1^H NMR spectrum (Figure S1h in Supplementary Materials contains a higher-resolution version of the figure); (**d**) GPC chromatogram; (**e**) TEM morphology image and particle size distribution histogram (Scale bar: 50 nm); (**f**) DLS particle size distribution curve.

The donor-acceptor conjugated polymer AT, consisting of benzodithiophene as the electron donor and the fused benzothiadiazole derivative AS_2_ as the electron acceptor, was successfully synthesized through Stille cross-coupling polymerization. The synthetic route is illustrated in Figure 2a. As shown in Figure 2b, the obtained polymer exhibited a deep ink-black appearance with a metallic luster, which was consistent with the formation of an extended π-conjugated polymer backbone.

The chemical structure was first confirmed by proton nuclear magnetic resonance (^1^H NMR) spectroscopy (Figure 2c). Compared with the sharp signals of the monomers, AT displayed broadened resonances characteristic of polymer formation. A broad aromatic signal distribution between δ 7.0 and 7.8 ppm was assigned to the aromatic protons of the benzodithiophene and benzothiadiazole units, together with the terminal cyanoindanone groups. The resonance at δ 2.91 ppm was attributed to the methylene protons adjacent to the fused aromatic rings, while the signals located at δ 1.2–1.6 ppm and δ 0.8–1.0 ppm corresponded to the alkyl-chain methylene and terminal methyl protons, respectively. These characteristic proton signals confirmed the successful incorporation of the designed structural units into the conjugated polymer backbone.

The molecular weight characteristics of AT were further evaluated by GPC (Figure 2d). The polymer exhibited a symmetric unimodal molecular-weight distribution without obvious low-molecular-weight impurities. The M_n_, M_w_, and PDI were determined to be 7341 g/mol, 14,267 g/mol, and 1.94, respectively, indicating a relatively uniform molecular-weight distribution.

The morphology and particle size of A_2_S-TT NPs were investigated by TEM and DLS. TEM images (Figure 2e) revealed that A_2_S-TT NPs possessed a uniform quasi-spherical morphology with well-defined particle boundaries and limited aggregation. Statistical analysis of multiple TEM images showed an average particle diameter of approximately 63 nm. The TEM element mapping diagram clearly demonstrates that all the various elements constituting A_2_S-TT NPs are uniformly distributed within a single nanoparticle, without obvious element aggregation, indicating that the elemental composition of the obtained sample is uniform (Figure S1b,c). Consistent with the TEM results, DLS analysis (Figure 2f) demonstrated a unimodal hydrodynamic size distribution with an average diameter of 81.29 nm, suggesting good colloidal uniformity. The slightly larger hydrodynamic diameter measured by DLS compared with TEM was attributed to the hydration layer surrounding the NPs in aqueous solution.

The A_2_S-TT NPs were characterized using an infrared spectrometer to obtain their infrared absorption spectrum (Figure S1a). The monomer TT exhibits the characteristic Sn-C vibration of -Sn(CH_3_)_3_ in the 500–800 cm^−1^ region; the monomer AS_2_ shows the characteristic absorption of -C≡N and C=O at approximately 2200 cm^−1^ and 1500–1600 cm^−1^, respectively, and the C-Br bond vibration signal can also be observed. The product AT retains the absorption peaks of -C≡N and C=O, while the characteristic C-Br and Sn-C absorption of the monomer completely disappear, confirming that TT and AS_2_ have reacted and preliminarily proving that the synthesis of AT is successful.

Collectively, these results demonstrate the successful construction of A_2_S-TT NPs with well-defined chemical structures and uniform nanoscale morphology. The favorable physicochemical characteristics, including appropriate particle size and narrow size distribution, provide a solid foundation for further research.

### 3.2. Physicochemical and Optical Properties of A_2_S-TT NPs

**Figure 3:**
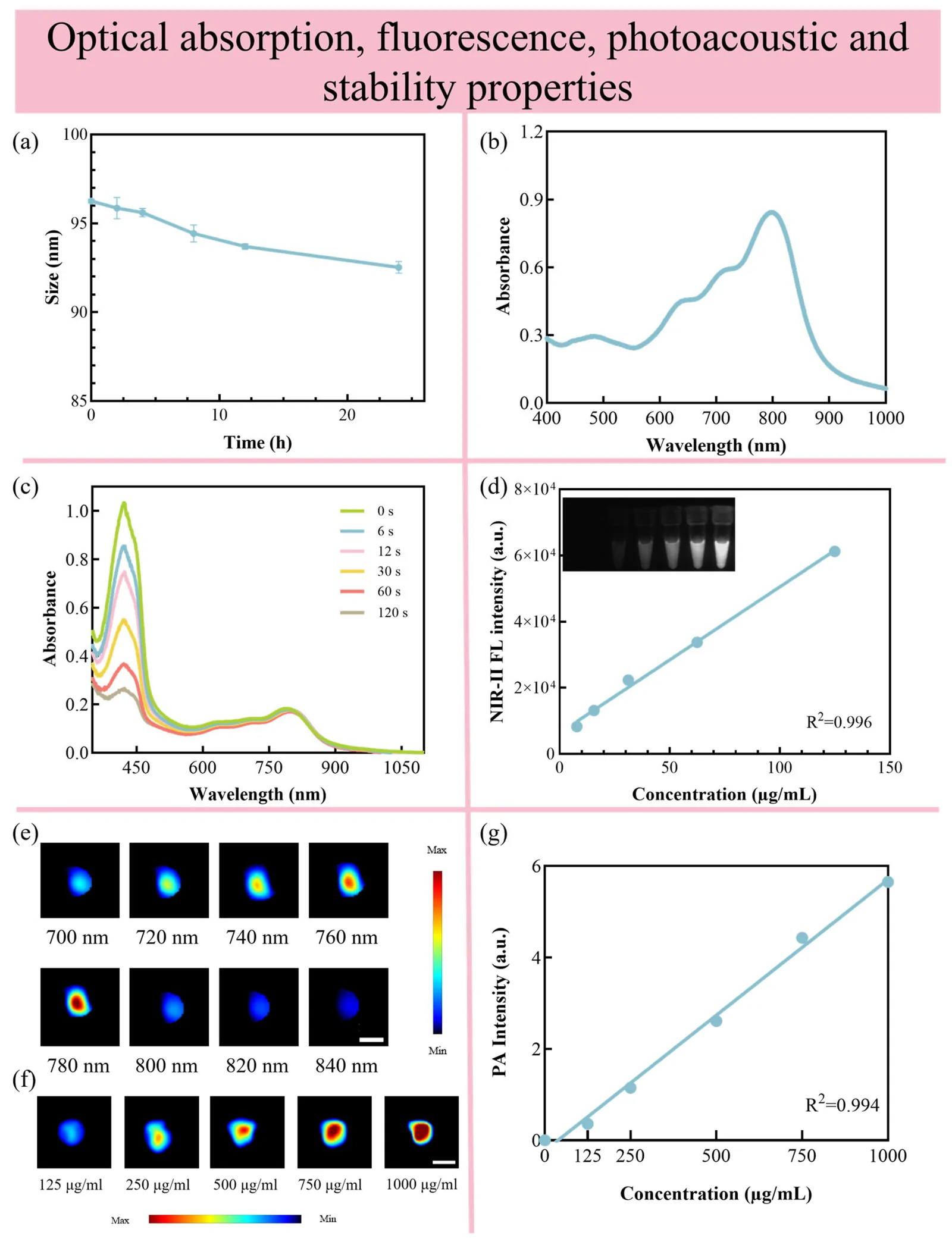
Photophysical properties and imaging capability. (**a**) The size stability of A_2_S-TT NPs over 24 h. Data were presented as mean values ± SD (*n* = 3); (**b**) UV-vis-NIR absorption spectrum; (**c**) DPBF decomposition absorption curves under irradiation; (**d**) NIR-II fluorescence images at different concentrations and corresponding intensity versus concentration linear fitting; (**e**) PA images at different excitation wavelengths (Scale bar: 1 mm); (**f**) PA images at different concentrations at 780 nm; (**g**) PA signal intensity versus concentration linear fitting (Scale bar: 1 mm).

The physicochemical stability of A_2_S-TT NPs was initially evaluated by monitoring the hydrodynamic diameter and potential variation over 24 h. The average particle size exhibited only a slight decline from 96.25 ± 0.0609 nm to 92.52 ± 0.1922 nm, corresponding to a variation of approximately 3.9% (Figure 3a). The average potential was within the range of −25 mV to −23 mV, with no significant attenuation (Figure S1e). This indicates that the size and zeta potential of the nanoparticles remain essentially unchanged within 24 h. The average particle size ranges from 89.77 nm to 93.05 nm, and the average zeta potential ranges from −24.93 mV to −23.45 mV across different media, suggesting minimal fluctuations in both average particle size and zeta potential (Figure S1d). Overall, A_2_S-TT NPs exhibits excellent colloidal stability.

The optical properties of A_2_S-TT NPs were subsequently investigated by UV-vis-NIR absorption spectroscopy (Figure 3b). A_2_S-TT NPs displayed a broad absorption profile in the near-infrared region, with a prominent absorption maximum at 789 nm. Compared with the relatively weak absorbance at 400 nm (0.286) and 1000 nm (0.064), the strong NIR absorption indicates the effective light-harvesting capability of A_2_S-TT NPs. Such a favorable NIR absorption characteristic is beneficial for photothermal conversion and provides the optical basis for NIR-based imaging and therapeutic applications.

The photodynamic activity of A_2_S-TT NPs was evaluated using DPBF as a singlet oxygen indicator (Figure 3c). Upon 808 nm laser irradiation, the characteristic DPBF absorption peak at 421 nm gradually decreased with prolonged irradiation time, accompanied by the sustained absorption of A_2_S-TT NPs at approximately 799 nm. This continuous reduction in DPBF absorbance confirmed the efficient generation of ROS by A_2_S-TT NPs under NIR irradiation, demonstrating their potential for PDT.

Furthermore, the NIR-II fluorescence imaging capability of A_2_S-TT NPs was assessed under 808 nm laser excitation (Figure 3d). The fluorescence intensity showed a strong linear correlation with A_2_S-TT NPs concentration, yielding an excellent calibration curve with an R^2^ value of 0.996. These results demonstrate the high sensitivity and quantitative capability of A_2_S-TT NPs as NIR-II fluorescent probes.

In addition, the PA performance of A_2_S-TT NPs was systematically investigated (Figure 3e–g). The photoacoustic signal intensity increased progressively with increasing A_2_S-TT NPs concentration, exhibiting a strong linear relationship with concentration (R^2^ = 0.994). And with the wavelength increasing from 700 nm to 840 nm, the PA signal intensity first rises and then declines, reaching its peak at 780 nm. These findings confirm the efficient photothermal-to-acoustic conversion capability of A_2_S-TT NPs and highlight their potential as a promising photoacoustic contrast agent for biomedical imaging applications.

### 3.3. In Vitro Antitumor Activity of A_2_S-TT NPs

The internalization process of A_2_S-TT NPs by 4T1 cells and their uptake kinetics at different time points were evaluated using cell uptake experiments. As shown in Figure S1f,g, the detection time points were set at 0, 2, 4, 8, and 12 h; the NIR-II fluorescence intensity of the cells increased over time, with an initial fluorescence intensity of 8.836 a.μ. at 0 h and a maximum value of 151.36 a.μ. reached at 12 h. This indicates that 4T1 cells effectively absorb A_2_S-TT NPs, and as time progresses, the amount of absorbed A_2_S-TT NPs increases, enabling these A_2_S-TT NPs to emit fluorescence under NIR-II fluorescence imaging.

**Figure 4:**
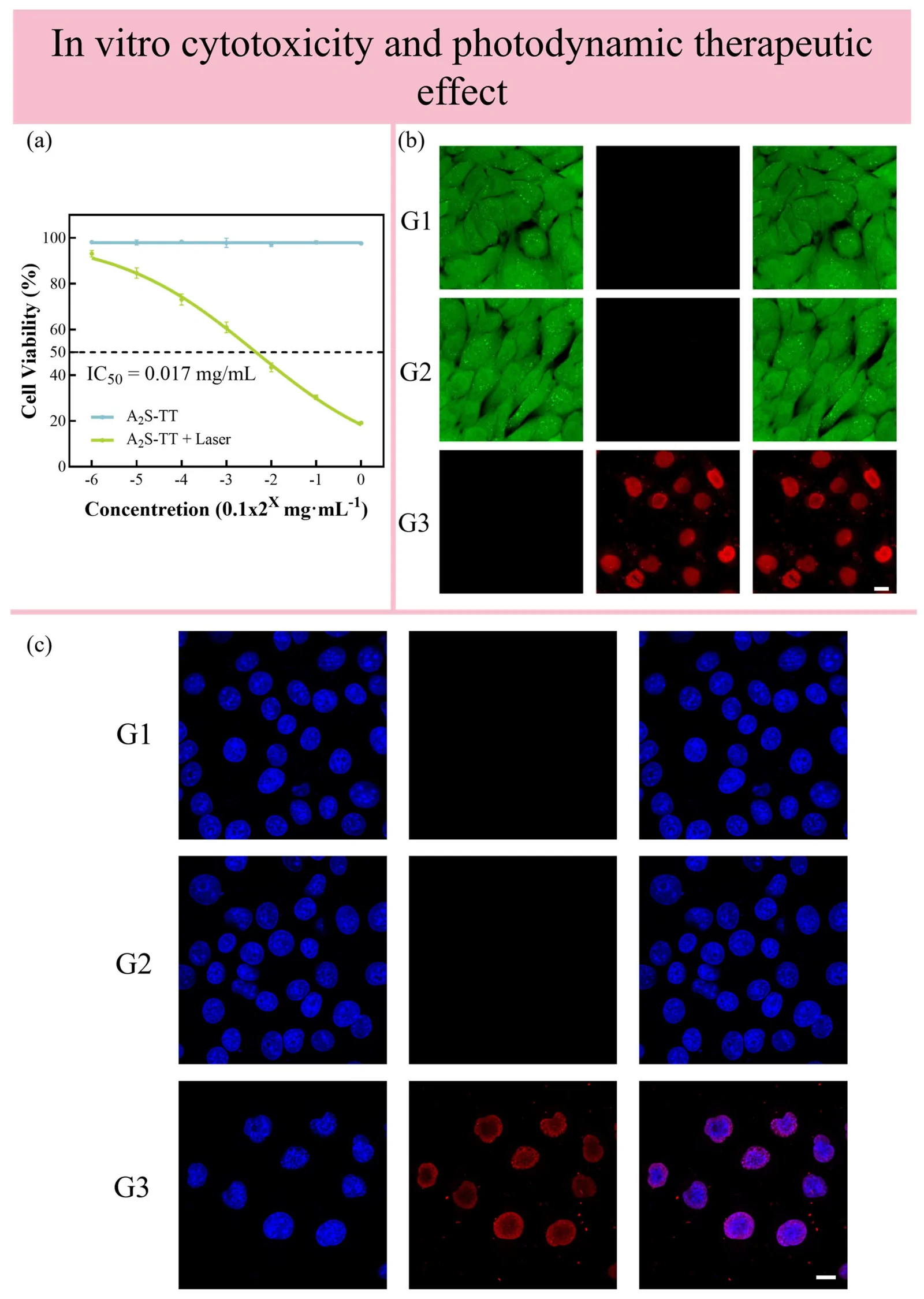
In vitro assessment of photodynamic cytotoxicity. (**a**) CCK-8 cell viability histogram with IC_50_; (**b**) Calcein-AM/PI live/dead staining images, with green fluorescence indicating viable cells and red fluorescence indicating dead cells (Scale bar: 10 μm); (**c**) DNA immunofluorescence images, with blue fluorescence indicating nuclei and red fluorescence indicating γ-H2AX, with merged images shown on the right (Scale bar: 10 μm). (Scale bar: 10 μm). G1: PBS; G2: A_2_S-TT NPs; G3: A_2_S-TT NPs plus laser.

The cytotoxicity of A_2_S-TT NPs with or without laser irradiation was evaluated in 4T1 cells using the CCK-8 assay. As shown in Figure 4a, A_2_S-TT NPs alone exhibited negligible cytotoxicity, with cell viability remaining above 97% even at a high concentration of 1 mg/mL, indicating the low cytotoxicity of A_2_S-TT NPs without irradiation. In contrast, upon 808 nm laser irradiation, A_2_S-TT NPs induced a concentration-dependent decrease in cell viability, which declined from 94.45% to 19.56% as the A_2_S-TT NPs concentration increased from 0.015625 mg/mL to 1 mg/mL. The calculated IC_50_ value was 0.017 mg/mL, demonstrating the potent photodynamic therapeutic effect of A_2_S-TT NPs upon NIR laser activation.

The phototherapeutic effect of A_2_S-TT NPs was further confirmed by live/dead cell staining (Figure 4b). 4T1 cells treated with PBS or A_2_S-TT NPs alone exhibited strong green fluorescence with negligible red fluorescence, indicating high cell viability. In contrast, cells treated with A_2_S-TT NPs followed by 808 nm laser irradiation displayed predominant red fluorescence, confirming efficient PDT-induced cell death.

To investigate DNA damage induced by A_2_S-TT NPs-mediated phototherapy, γ-H2AX immunofluorescence staining was performed (Figure 4c). Compared with the PBS and A_2_S-TT NPs groups, which showed only weak nuclear γ-H2AX signals, the A_2_S-TT NPs plus laser irradiation group exhibited pronounced red fluorescence in the nuclei, indicating substantial DNA double-strand break formation. These results demonstrated that A_2_S-TT NPs possess excellent biocompatibility in the dark while effectively inducing tumor cell apoptosis and DNA damage under NIR laser irradiation.

Taken together, A_2_S-TT NPs exhibit low dark toxicity and achieve efficient NIR laser-triggered tumor cell killing accompanied by DNA damage induction, highlighting their potential for phototherapeutic applications.

### 3.4. Mechanistic Investigation of A_2_S-TT NPs-Mediated PDT-Induced Cell Death

**Figure 5:**
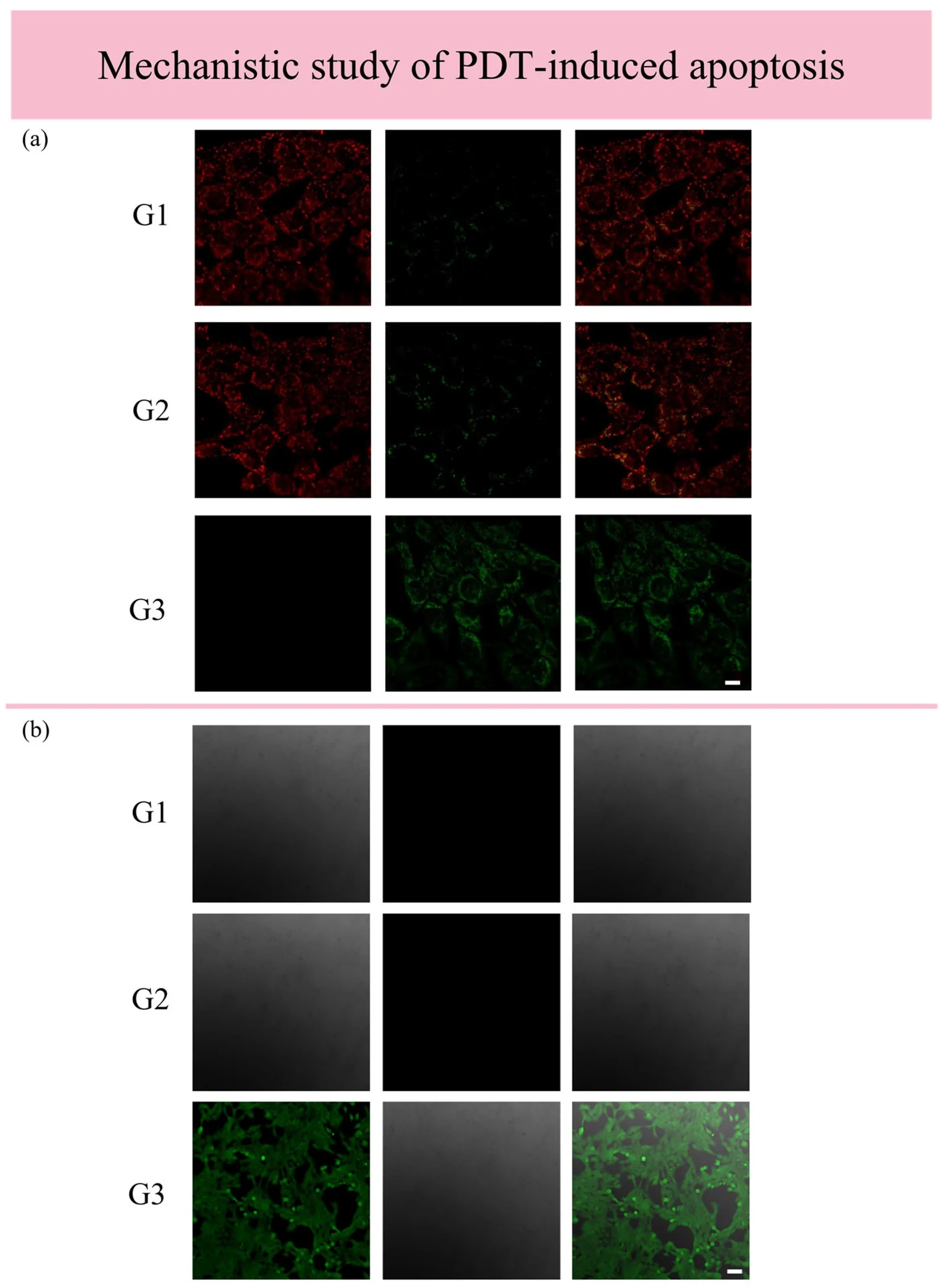
Investigation of PDT-induced apoptotic signaling. (**a**) JC-1 mitochondrial membrane potential staining images, with red fluorescence indicating JC-1 aggregates and higher mitochondrial membrane potential, and green fluorescence indicating JC-1 monomers and lower mitochondrial membrane potential (Scale bar: 10 μm); (**b**) DCFH-DA intracellular ROS fluorescence images, with green fluorescence indicating intracellular ROS (Scale bar: 50 μm). G1: PBS; G2: A_2_S-TT NPs; G3: A_2_S-TT NPs plus laser.

The effects of A_2_S-TT NPs with or without laser irradiation on mitochondrial membrane potential changes in 4T1 cells were evaluated using JC-1 staining. JC-1 is a cationic fluorescent dye that accumulates in the mitochondrial matrix in a potential-dependent manner. In healthy cells, JC-1 forms aggregates and emits red fluorescence, whereas mitochondrial depolarization causes JC-1 monomers to accumulate, resulting in enhanced green fluorescence. As shown in Figure 5a, 4T1 cells treated with PBS and A_2_S-TT NPs alone exhibited strong red fluorescence, indicating preserved mitochondrial membrane potential. In contrast, cells treated with A_2_S-TT NPs followed by 808 nm laser irradiation showed markedly increased green fluorescence accompanied by reduced red fluorescence, suggesting significant mitochondrial membrane depolarization. These results indicate that A_2_S-TT NPs-mediated phototherapy induces mitochondrial dysfunction, which contributes to tumor cell death.

Intracellular ROS generation was further assessed using the DCFH-DA fluorescent probe (Figure 5b). DCFH-DA can penetrate cell membranes and is hydrolyzed intracellularly to form non-fluorescent DCFH, which is subsequently oxidized by ROS to generate fluorescent DCF. 4T1 cells in the PBS and A_2_S-TT NPs groups showed negligible green fluorescence, indicating minimal ROS accumulation in the absence of irradiation. In contrast, cells treated with A_2_S-TT NPs under 808 nm laser irradiation exhibited intense green fluorescence, demonstrating efficient intracellular ROS generation upon NIR activation. These findings confirm that A_2_S-TT NPs function as an effective photosensitizing nanoplatform, inducing ROS-mediated oxidative stress and mitochondrial dysfunction under laser irradiation, thereby contributing to phototherapy-induced tumor cell killing.

Mechanistic investigations revealed that A_2_S-TT NPs-mediated PDT induces tumor cell death through a cascade of ROS-driven mitochondrial dysfunction. NIR-triggered A_2_S-TT NPs function as an efficient photosensitizing nanoplatform, wherein amplified oxidative stress instigates mitochondrial impairment, thereby propagating ROS-mediated apoptotic signalling and ultimately culminating in potent tumoricidal efficacy.

### 3.5. In Vivo NIR-II Fluorescence/PA and Biodistribution of A_2_S-TT NPs

**Figure 6:**
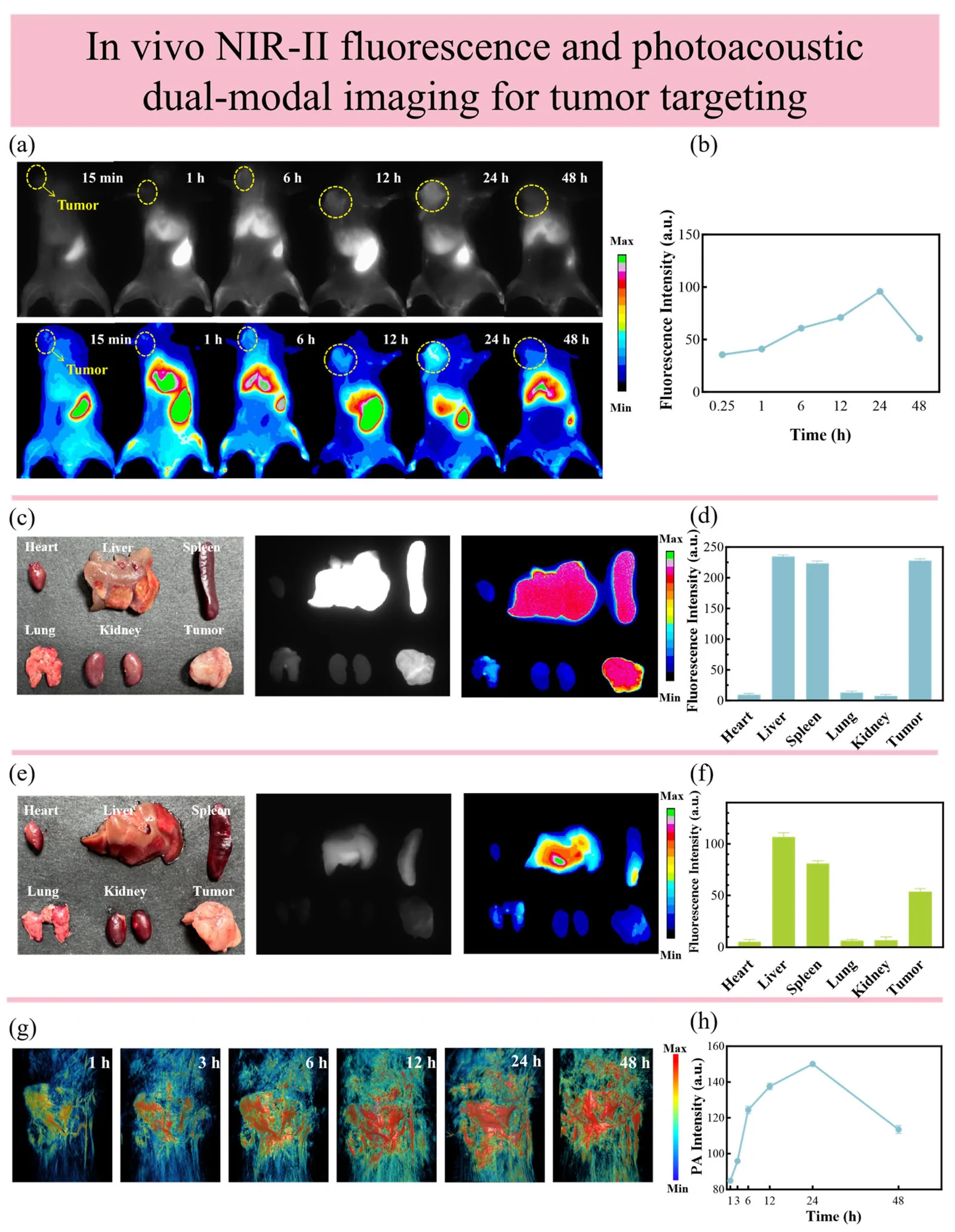
NIR-II fluorescence and PA of tumor accumulation. (**a**) Whole-body NIR-II fluorescence time-course images; (**b**) Tumor fluorescence intensity quantification curve; (**c**) Ex vivo organ imaging at 24 h; (**d**) Quantitative fluorescence histogram at 24 h; (**e**) Ex vivo imaging at 48 h; (**f**) Quantitative histogram at 48 h; (**g**) PA time-course images; (**h**) PA signal quantification curve. Yellow dashed circles indicate the tumor regions. The pseudocolor scale represents signal intensity, with blue indicating lower intensity and red indicating higher intensity. Data were presented as mean values ± SD (*n* = 3).

To investigate the in vivo tumor accumulation behavior of A_2_S-TT NPs, NIR-II fluorescence imaging was performed in 4T1 tumor-bearing mice after intravenous administration. As shown in Figure 6a, the tumor fluorescence signal gradually increased over time and reached the maximum intensity at 24 h post-injection, indicating efficient accumulation of A_2_S-TT NPs within tumor tissues. Although the signal decreased at 48 h, a considerable fluorescence intensity was still retained at the tumor site, suggesting prolonged tumor retention of A_2_S-TT NPs. Quantitative analysis of tumor fluorescence intensity (Figure 6b) confirmed a time-dependent accumulation pattern, with the fluorescence intensity increasing from 36.59 ± 1.27 at 0.25 h to 96.37 ± 1.25 at 24 h, followed by a moderate decrease to 51.83 ± 1.22 at 48 h. These results demonstrate that A_2_S-TT NPs possess favorable tumor accumulation and retention properties, providing an appropriate imaging window for subsequent phototherapy.

The biodistribution of A_2_S-TT NPs was further evaluated by ex vivo NIR-II fluorescence imaging of major organs and tumors at 24 and 48 h post-injection (Figure 6c,d). At 24 h, the tumor exhibited a strong fluorescence signal compared with major organs, confirming effective tumor accumulation. At 48 h, substantial fluorescence remained in the tumor, while relatively strong signals were observed in the liver and spleen, suggesting the involvement of the reticuloendothelial system in A_2_S-TT NPs clearance. These findings indicate that A_2_S-TT NPs exhibited prolonged tumor retention and a characteristic biodistribution profile after systemic administration.

The PA capability of A_2_S-TT NPs was subsequently evaluated in tumor-bearing mice (Figure 6e–h). Following intravenous injection, the PA signal at the tumor site progressively increased and reached its maximum at 24 h, consistent with the NIR-II fluorescence imaging results. Quantitative analysis revealed that the PA signal increased from 84.88 ± 1.35 at 1 h to 150.11 ± 1.40 at 24 h, demonstrating enhanced tumor-specific imaging contrast. Although the signal slightly declined at 48 h, a considerable PA signal was still maintained, indicating the favorable tumor retention capability of A_2_S-TT NPs.

Together, these results demonstrate that A_2_S-TT NPs enabled complementary NIR-II fluorescence and PA with high sensitivity, prolonged tumor accumulation, and sustained imaging signals, supporting its potential as an imaging-guided phototherapeutic nanotheranostics.

### 3.6. Imaging-Guided PDT and Biosafety Evaluation of A_2_S-TT NPs In Vivo

**Figure 7:**
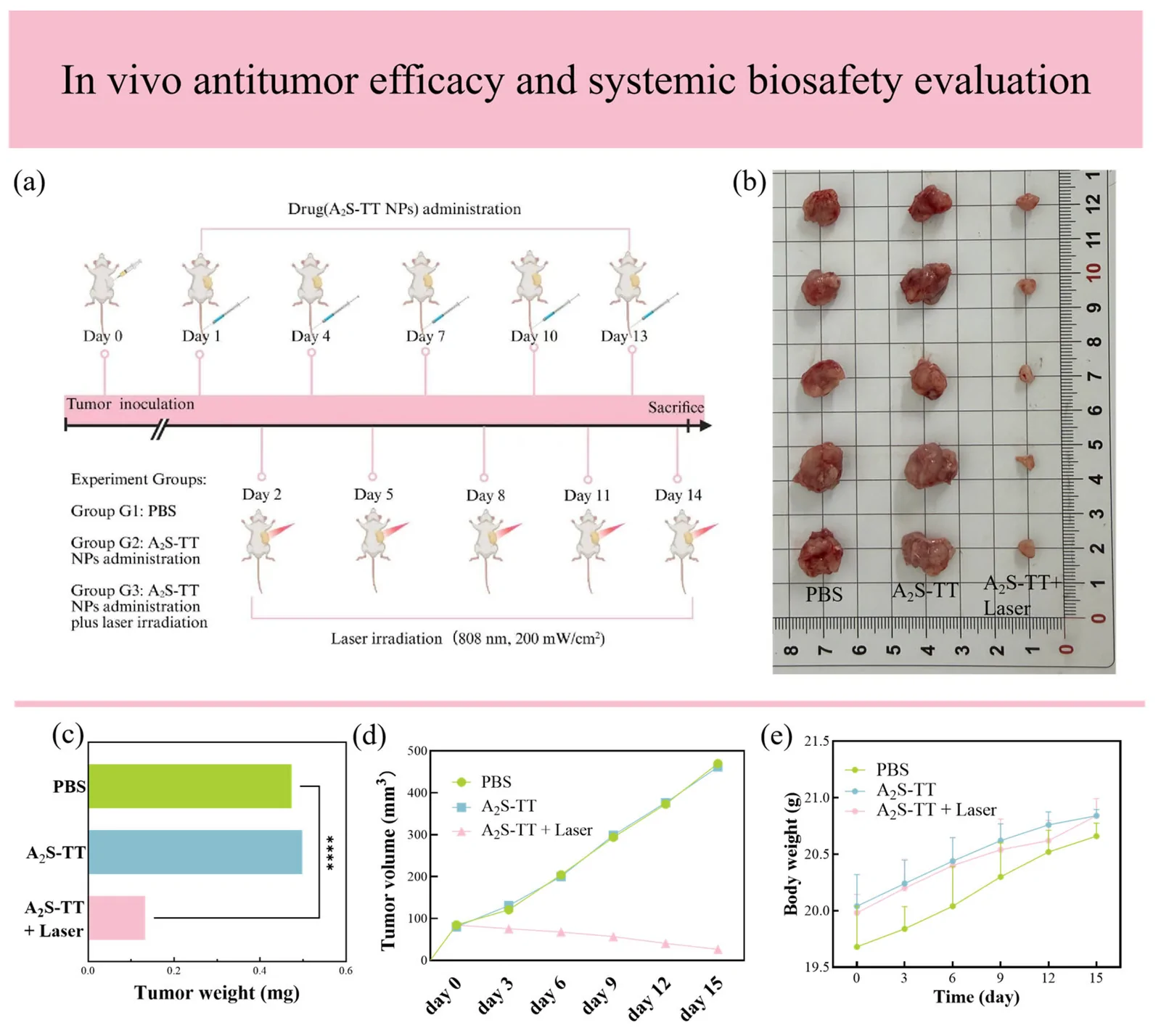
Antitumor efficacy and biocompatibility assessment in vivo. (**a**) Schematic diagram of drug administration and treatment plan for in vivo animal experiments; (**b**) Ex vivo tumor photographs; (**c**) Tumor weight histogram (**** *p* < 0.0001); (**d**) Tumor volume growth curves; (**e**) Body weight change curves.

The in vivo antitumor activity of A_2_S-TT NPs was evaluated in 4T1 tumor-bearing mice, with the experimental protocol illustrated in Figure 7a. At the end of the treatment, the ex vivo tumor photographs (Figure 7b) visually demonstrated that the tumor volume in the A_2_S-TT NPs + laser group was significantly smaller compared with the PBS and A_2_S-TT NPs groups. Tumor weight statistics (Figure 7c) revealed that the weights in the PBS, A_2_S-TT NPs, and A_2_S-TT NPs + laser groups were 0.4734 ± 0.0362 g, 0.4979 ± 0.0284 g, and 0.1325 ± 0.0098 g, respectively. Tumor volume growth curves (Figure 7d) indicated that the initial volumes were comparable among groups; however, by the end of the treatment, the tumor volumes in the PBS and A_2_S-TT NPs groups increased to 469.67 ± 36.54 mm^3^ and 461.70 ± 19.54 mm^3^, respectively, while the tumor volume in the A_2_S-TT NPs + laser group was markedly reduced to 26.11 ± 3.92 mm^3^, achieving a tumor inhibition rate of approximately 72.0%. During the treatment period, the body weights of mice in all groups remained stable (Figure 7e), preliminarily indicating that the therapy induced no significant systemic toxicity.

**Figure 8:**
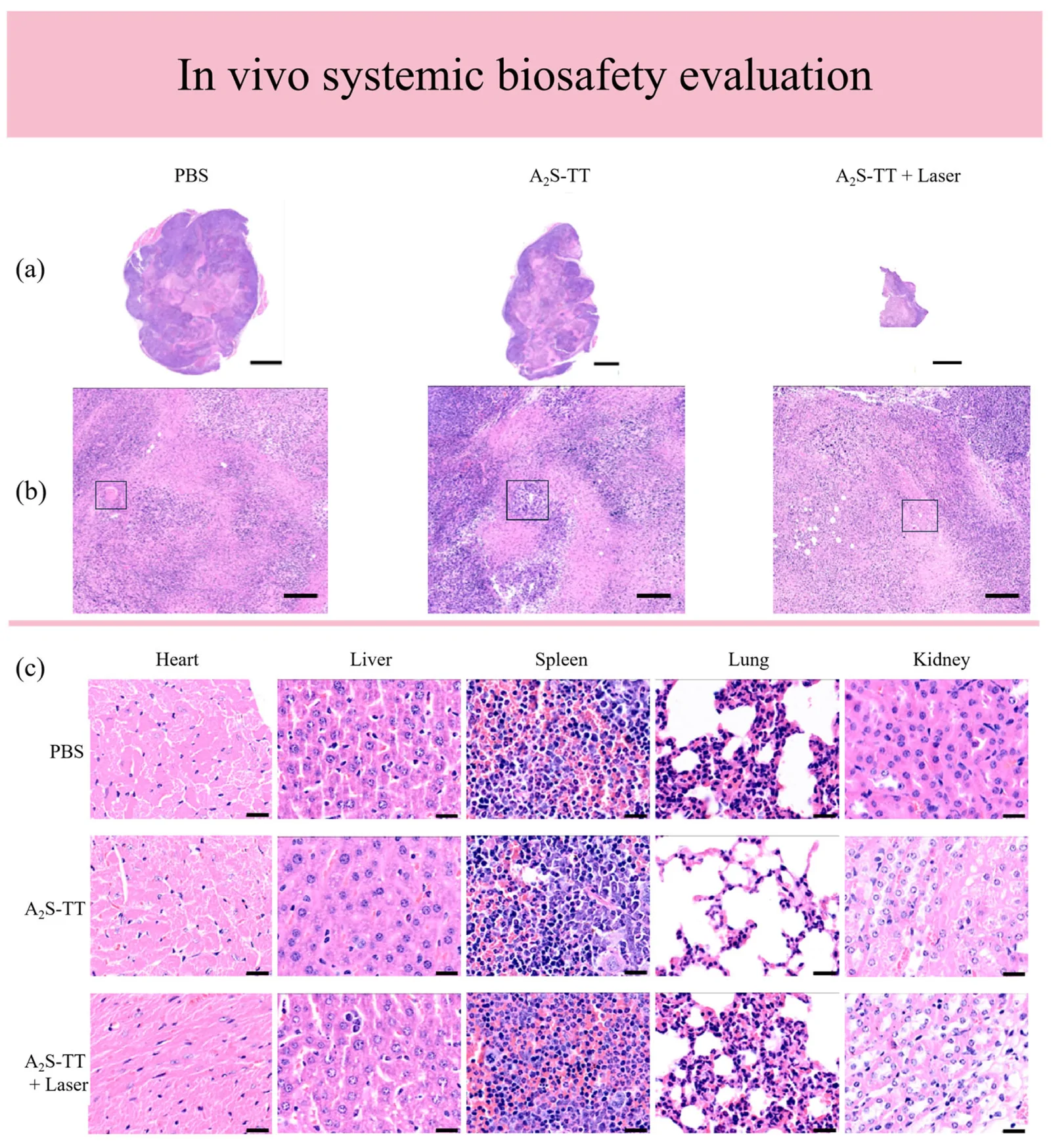
(**a**) Representative images of full-view H&E-stained tissue slices (Scale bar: 2 mm); (**b**) Characteristic mammary duct regions (Scale bar: 200 μm); (**c**) Histopathological H&E staining of key visceral organs, including the heart, liver, spleen, lung, and kidney (Scale bar: 20 μm). Data were presented as mean values ± SD (*n* = 5).

Histopathological results further confirmed the antitumor efficacy of the A_2_S-TT NPs + laser group. As shown in the full-view H&E-stained sections (Figure 8a), the tumor tissues in the PBS and A_2_S-TT NPs groups were dense with active cell growth, whereas the tumor tissues in the A_2_S-TT NPs + laser group exhibited extensive loose areas and necrotic regions. When observing the characteristic mammary duct regions at higher magnification (Figure 8b), the A_2_S-TT NPs + laser group exhibited obvious nuclear pyknosis, fragmentation, and severe disruption of cellular structures, indicating that PDT induced potent tumor cell killing effects. In contrast, the cells in the control groups displayed relatively intact morphology and tight arrangement. Furthermore, H&E staining of major organs, including the heart, liver, spleen, lungs, and kidneys (Figure 8c), revealed no obvious pathological abnormalities or tissue damage in any treatment group, including the A_2_S-TT NPs + laser group, further confirming the excellent in vivo biosafety of A_2_S-TT NPs.

Overall, A_2_S-TT NPs achieve efficient tumor growth inhibition through NIR-II fluorescence/PA-guided PDT and exhibit minimal systemic toxicity, demonstrating their potential as a multifunctional nanoplatform for integrated tumor imaging and therapy.

## 4. Discussion

This study developed a novel PDT platform guided by PA/NIR-II dual-modal imaging using the donor-acceptor conjugated polymer A_2_S-TT NPs, achieving an integrated approach to diagnosis and treatment. PA imaging provides high-resolution information about deep tissues [25,26], while NIR-II fluorescence imaging exhibits high sensitivity and tissue penetration capability [27,28]; the complementary advantages of these two modalities enable the platform to achieve precise tumor localization, dynamic monitoring, and therapeutic visualization [29,30]. Under dual-modal PA + NIR-II imaging guidance, A_2_S-TT NPs is accurately enriched at tumor sites and efficiently generate ROS upon irradiation with 808 nm laser light, significantly enhancing PDT efficacy [31]. Additionally, in vitro and in vivo biosafety evaluations demonstrated that A_2_S-TT NPs exhibit excellent biocompatibility, with no significant systemic toxicity or major organ damage observed at therapeutic doses, indicating promising application potential. Overall, this study establishes an integrated diagnostic and therapeutic strategy combining precise imaging, efficient treatment, and superior safety, offering a novel approach for precision PDT in TNBC.

Although this study has demonstrated the application value of A_2_S-TT NPs in imaging-guided PDT, certain limitations remain. First, only a preliminary in vivo safety evaluation was conducted, with results indicating no significant cytotoxicity; however, its long-term metabolic and clearance pathways in vivo require further investigation. Second, the key protein pathways and regulatory networks involved in A_2_S-TT NPs-induced cell death have not yet been fully elucidated. Future studies integrating transcriptomics and proteomics may help unravel its antitumor mechanisms, providing a theoretical basis for optimizing diagnostic and therapeutic platforms.

From a clinical translation perspective, the safety and efficacy of A_2_S-TT NPs can be further validated in animal models with higher clinical relevance, followed by systematic pharmacokinetic studies and large-scale production development, thereby laying the foundation for its translation into a clinical diagnostic and therapeutic platform [32,33].

## 5. Conclusions

In summary, this study developed the A_2_S-TT NPs nanotheranostics as the core functional scaffold, PA–NIR-II dual-modal imaging as the imaging component, and PDT as the therapeutic method to establish an integrated diagnostic and therapeutic platform for breast cancer. The core A_2_S-TT NPs scaffold exhibits excellent near-infrared response properties, high biosafety, and effective tumor-targeted accumulation capability. The PA–NIR-II dual-modal imaging enables precise localization and dynamic monitoring of tumor tissues. PDT—allows the A_2_S-TT NPs, which are enriched at the tumor lesion site, to efficiently release ROS upon excitation with an 808 nm laser, thereby inducing apoptosis in breast cancer cells and achieving precise treatment of TNBC. This study confirms that the NIR-II plus PA dual-modal system-mediated A_2_S-TT NPs possess superior imaging capabilities and precise targeting efficacy for TNBC, positioning it as a promising integrated photodynamic diagnosis and therapy nanotheranostics with exceptional biosafety and targeted therapeutic potential in future clinical applications.

## Data Availability

The original contributions presented in this study are included in the article/Supplementary Materials. Further inquiries can be directed to the corresponding authors.

## Supplementary Materials

The following supporting information can be downloaded at: https://www.mdpi.com/article/10.3390/pharmaceutics18091144/s1, Figure S1: (**a**) FT-IR spectra of AS_2_, TT and A_2_S-TT NPs; (**b**) EDS energy-dispersive X-ray spectrum of A_2_S-TT NPs; (**c**) TEM image and corresponding elemental mapping of A_2_S-TT NPs (Scale bar: 100 nm); (**d**) Hydrodynamic diameter (HD) and zeta-potential of A_2_S-TT NPs incubated in different media; (**e**) Time-dependent changes in HD and zeta-potential of A_2_S-TT NPs; (**f**) NIR-II fluorescence intensity of 4T1 cells after incubation with A_2_S-TT NPs for different times. Data are presented as mean ± SD; (**g**) NIR-II fluorescence imaging images of 4T1 cells co-incubated with A_2_S-TT NPs for different time points (0, 2, 4, 8, 12 h); (**h**) ^1^H NMR spectrum.

## Abbreviations

The following abbreviations are used in this manuscript:

NIR-II	Near-infrared II
PA	Photoacoustic
TNBC	Triple-negative breast cancer
HER2	Human epidermal growth factor receptor 2
MRI	Magnetic resonance imaging
PDT	Photodynamic therapy
ROS	Reactive oxygen species
Mn	Number-average molecular weight
Mw	Weight-average molecular weight
TEM	Transmission electron microscope
DLS	Dynamic light scattering
DPBF	1,3-Diphenylisobenzofuran
DMEM	Dulbecco’s modified eagle medium
FBS	Fetal bovine serum
PS	Penicillin-streptomycin solution
EDTA	Ethylenediaminetetraacetic acid
DCFH-DA	2′,7′-Dichlorodihydrofluorescein diacetate
DCF	2,7-Dichlorofluorescein
H&E	Hematoxylin and eosin staining
TIR	Tumor inhibition rate
DMSO	Dimethyl sulfoxide
GPC	Gel permeation chromatography
THF	Tetrahydrofuran
^1^H NMR	Protonnuclear magnetic resonance
A_2_S-TT NPs	A_2_S-TT nanoparticles
DMF	Dimethylformamidex
