# Supplementary material for: Programmable Organic A2S-TT NPs for NIR-II/Photoacoustic Imaging-Enabled Precision Therapy
Source: Pharmaceutics. 2026 Sep 10;18(9):1144. doi: 10.3390/pharmaceutics18091144 (PMC13610734; doi:10.3390/pharmaceutics18091144)
Supplement: Supplementary file 1 [file pharmaceutics-18-01144-s001.zip › pharmaceutics-4501541-supplementary.pdf]

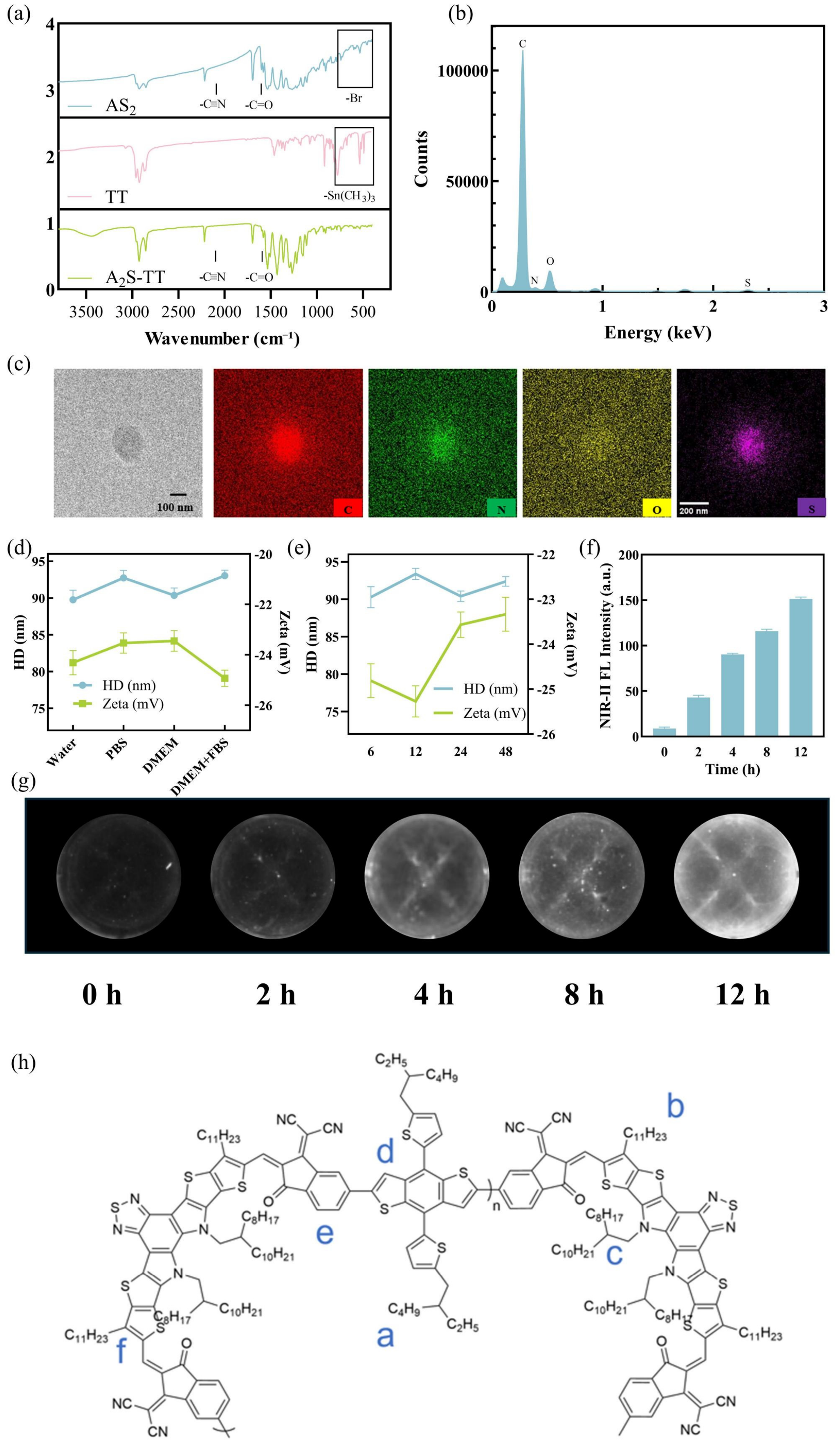

(a)
AS2
-C≡N
-C=O
-Br
TT
-Sn(CH3)3
A2S-TT
Wavenumber (cm⁻¹)
(b)
Counts
Energy (keV)
C
N
O
S
(c)
100 nm
200 nm
C
N
O
S
(d)
HD (nm)
Zeta (mV)
Water
PBS
DMEM
DMEM+FBS
(e)
HD (nm)
Zeta (mV)
(f)
NIR-II FL Intensity (a.u.)
Time (h)
(g)
0 h
2 h
4 h
8 h
12 h
(h)
a
b
c
d
e
f

Figure S1. (**a**) FT-IR spectra of AS_2, TT and A_2S-TT NPs; (**b**) EDS energy-dispersive X-ray spectrum of A_2S-TT NPs; (**c**) TEM image and corresponding elemental mapping of A_2S-TT NPs (Scale bar: 100 nm); (**d**) Hydrodynamic diameter (HD) and zeta-potential of A_2S-TT NPs incubated in different media; (**e**) Time-dependent changes in HD and zeta-potential of A_2S-TT NPs; (**f**) NIR-II fluorescence intensity of 4T1 cells after incubation with A_2S-TT NPs for different times. Data are presented as mean ± SD; (**g**) NIR-II fluorescence imaging images of 4T1 cells co-incubated with A_2S-TT NPs for different time points (0, 2, 4, 8, 12 h); (**h**) ^1H NMR spectrum.
